# Refinement of IntelliCage protocols for complex cognitive tasks through replacement of drinking restrictions by incentive-disincentive paradigms

**DOI:** 10.3389/fnbeh.2023.1232546

**Published:** 2023-11-16

**Authors:** Xueqian Ma, Beatrice Schildknecht, Adrian C. Steiner, Irmgard Amrein, Martina Nigri, Giulia Bramati, David P. Wolfer

**Affiliations:** ^1^Department of Health Sciences and Technology, Institute of Human Movement Sciences and Sport, ETH, Zürich, Switzerland; ^2^Faculty of Medicine, Institute of Anatomy, University of Zürich, Zürich, Switzerland

**Keywords:** home cage monitoring, incentive learning, disincentive learning, appetitive learning, aversive learning, animal welfare, 3R principles, C57BL/6 N mice

## Abstract

The IntelliCage allows automated testing of cognitive abilities of mice in a social home cage environment without handling by human experimenters. Restricted water access in combination with protocols in which only correct responses give access to water is a reliable learning motivator for hippocampus-dependent tasks assessing spatial memory and executive function. However, water restriction may negatively impact on animal welfare, especially in poor learners. To better comply with the 3R principles, we previously tested protocols in which water was freely available but additional access to sweetened water could be obtained by learning a task rule. While this purely appetitive motivation worked for simple tasks, too many mice lost interest in the sweet reward during more difficult hippocampus-dependent tasks. In the present study, we tested a battery of increasingly difficult spatial tasks in which water was still available without learning the task rule, but rendered less attractive either by adding bitter tasting quinine or by increasing the amount of work to obtain it. As in previous protocols, learning of the task rule provided access to water sweetened with saccharin. The two approaches of dual motivation were tested in two cohorts of female C57BL/6 N mice. Compared to purely appetitive motivation, both novel protocols strongly improved task engagement and increased task performance. Importantly, neither of the added disincentives had an adverse impact on liquid consumption, health status or body weight of the animals. Our results show that it is possible to refine test protocols in the IntelliCage so that they challenge cognitive functions without restricting access to water.

## Introduction

Traditionally, behavioral analysis of laboratory animals is performed using batteries of tests that are conducted in a specific experimental setup. To name but a few, these include simple conditioning chambers (Heron and Skinner, 1939), the nesting (Deacon, 2006a) and burrowing (Deacon, 2006b) tests to assess the intactness of complex instinctive behaviors or the open field test to quantify emotionality and spontaneous activity (Hall, 1934). An especially broad range of experiments has been developed to explore different facets of memory (see Ghafarimoghadam et al., 2022, for a comprehensive review), including the Morris water maze (Morris et al., 1982; D’Hooge and De Deyn, 2001) and Barnes maze tasks (Barnes, 1979) as tests of spatial learning capability.

While these tests remain crucial to phenotyping new animal models of neurological diseases, they share a number of drawbacks: Animals are exposed to an unfamiliar and thus stressful environment, they need to be separated from their cage mates and the necessity for human intervention introduces undesirable variability across laboratories and experimenters (Crabbe et al., 1999; Chesler et al., 2002; Wahlsten et al., 2003).

To address this issue, systems that automatically monitor animals in their home cage environment have been developed, using various mechanisms such as infrared beams, video tracking and operant task machines inside the cage (Voikar and Gaburro, 2020).

**Figure 1:**
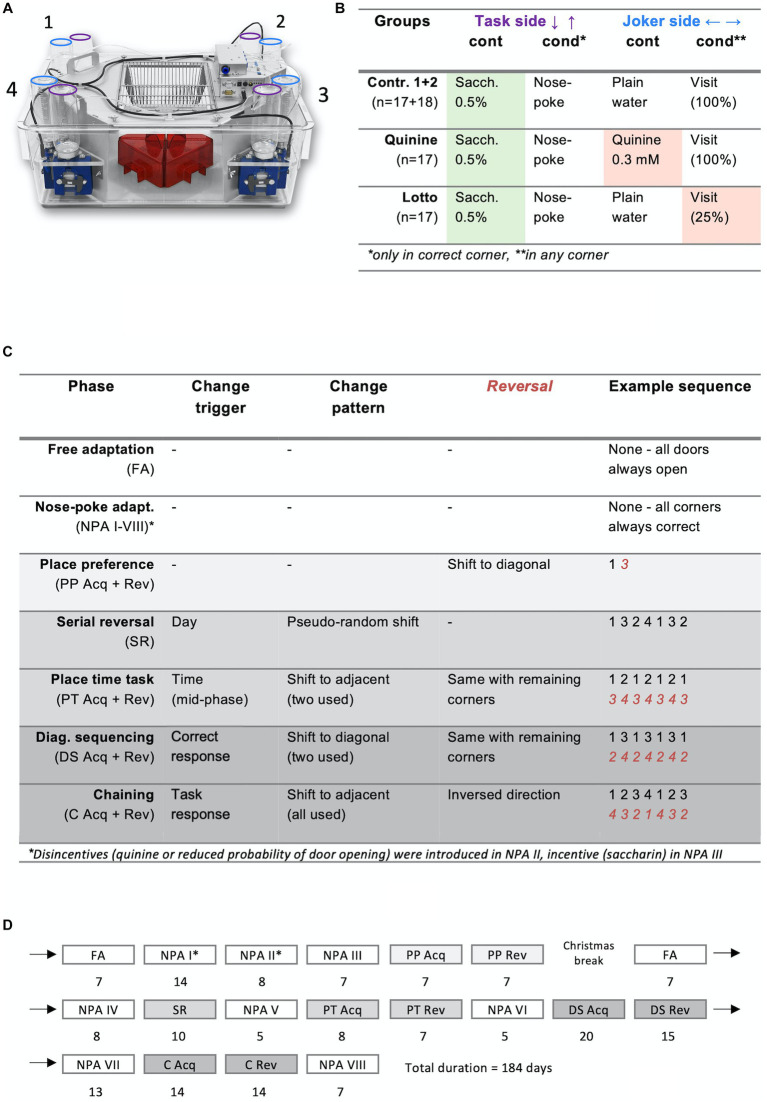
Overview of study design. **(A)** Photograph of an IntelliCage: Red rodent house in the middle, learning corners with drinking bottles on the side. Purple = task side bottles, blue = joker side bottles. Note: The added extension cage is not shown here. **(B)** Programming and content of drinking bottles. Task sides opened for 3 s after a nose poke, whereas joker sides opened for 3 s on every corner visit. Joker sides were made unattractive using either bitter tasting quinine solution or decreased opening probability in experiment groups. Task sides were made more attractive with saccharin solution for all groups. Task side bottles were only available in correct corners, whereas joker side bottles were accessible in all four corners. **(C)** Phases. Shading reflects difficulty. Reversal sequences in red italic numbers. **(D)** Timeline. Shading again reflects difficulty. ^*^Note that modifications were gradually introduced in NPA I-III. NPA I, No disincentive or incentive; NPA II, Only disincentive; NPA III-VIII, Both disincentive and incentive (saccharin).

The IntelliCage system (New Behavior AG, TSE systems, see Figure 1A for a photograph of the apparatus) remains the most flexible of these concepts (Kiryk et al., 2020; Lipp et al., 2023) and offers the advantage of social housing. It uses transponder-based radio-frequency identification and four learning corners that each contain two operant conditioning walls with a motorized door regulating access to the nipple of a drinking bottle. Although the inputs required from the animals are simple (“visits” to the learning corners are measured by presence of body heat and transponder signal, “nose pokes” on the operant doors are recorded through infrared beams and “licks” at the bottle nipples are registered by contact sensors), adjustments to the spatial and temporal sequence of correct doors and various complications such as light indicators, unpleasant air-puffs or olfactory cues allow for an extremely broad range of possible experiments to study many aspects of rodent behavior.

A disadvantage of the IntelliCage system is the fact that in most learning tasks, drinking is provided only if tasks are correctly completed, which means that thirst is the primary motivational driver. Drinking sessions are usually limited to 2–4 intervals of 1–2 h a day to improve learning. Under these circumstances, animals that learn poorly or insist on wrong choice patterns are at risk for dehydration. Finding a way to replace drinking restrictions is an ethically desirable goal, as it reduces suffering imposed on laboratory animals through refinement of the experimental process in accordance with the last element of the 3-R principles “replace, reduce, refine” (Russell, 1960).

Concerns regarding the impact of liquid deprivation on animal welfare are increasing. In Switzerland, water deprivation of mice is only considered mild if it lasts for less than 12 h, deprivation periods of 12–24 h are classified as moderate constraint (severity grade 2 according the Swiss Federal Food Safety and Veterinary Office FSVO; FSVO, 2018). In the recently proposed d’Isa-Gerlai rating scale for the impact of behavioral tests on animal welfare, on a 12-level scale ranging from A (animal-friendly) to L (lethal), water deprivation with duration > 9 h has been rated H (d’Isa and Gerlai, 2023).

One way to replace thirst as the primary learning incentive is to make plain water constantly available, but reward correct task completion with a liquid of more attractive taste. C57BL/6 mice are known to prefer water containing nutritive sugars as well as non-nutritive sweeteners (Sclafani et al., 2010). In a previous study (Bramati et al., 2023), we showed that appetitive learning based solely on preference for sweetened water can work sufficiently well in simple learning tasks, but is insufficient for reliably providing the stronger motivation needed to learn more complex tasks.

For this reason, we sought a way to improve motivation while still constantly providing drinking water to all animals. To do so, we decided to add a disincentive to the constantly available water. In this study, we compare two ways to achieve this: the addition of bitter tasting quinine, which has been shown to be disliked by mice (Masamoto et al., 2020; Kahnau et al., 2023) and the introduction of a “gambling” mechanism that denied access to plain water in 75% of responses for plain water, which increases the amount of effort needed to obtain the same volume of water. Our aim was to expand the range of tasks that can be successfully employed in a reward learning paradigm and explore the limits of our new approach. To this end, we tested our methods in a series of increasingly difficult place-learning tasks and compared them to purely appetitive motivation.

## Materials and methods

All experimental procedures were approved by the Cantonal Veterinary Office of Zurich (License No. 060/2021).

### Animals

A total of 69 female C57BL/6 N mice (Charles River Laboratories, Germany) were used in the study. Their age was 2 months on arrival in our facility and they were housed in a 12:12-h reversed light–dark cycle (lights off 08:00–20:00). Radio frequency identification transponders were implanted subcutaneously into the neck region under inhalation anesthesia with isoflurane. Recovery from the implantation procedure and stability of transponder placement was observed during a 7-day period. Mice were introduced to IntelliCages at the age of 3 months, which is when experiments started.

Animals were randomly assigned to two experimental and two control groups. Control groups were subjected to a series of appetitive-learning tasks that included saccharin alone as incentive, while experiment groups were exposed to the combined incentive-disincentive paradigm, with either quinine or decreased reward probability as the disincentive.

Mice that scored fewer than 100 licks daily (which indicates a clear drop from the usually observed baseline of approximately 1,000 licks a day) were temporarily transferred to a separate cage, where they were provided with an *ad libitum* supply of drinking water. If sufficient drinking did not resume after a few days, they were excluded from the study and placed back into normal housing. In total, 3 animals were temporarily transferred to separate cage and returned, whereas 13 were permanently excluded (6 from neutral groups and 7 from disincentive groups).

### Nomenclature

The four learning corners are numbered in a clockwise manner, starting from the upper left (Figure 1A). Each learning corner has two operant doors. The operant doors on the long edges of the cage are termed “Task side” doors (where Saccharin could be obtained for correct responses), whereas the ones located on the short edges are called “Joker side” doors. The initial stage of each learning task is called “acquisition stage,” and the following phase with inversed conditions is termed “reversal stage.” For the sake of brevity, groups are named after the joker side doors (control groups were termed “neutral” and incentive/disincentive experiment groups were called “disincentive”). “Lotto” and “Quinine” were chosen as short names for, respectively, the experiment with the gambling condition and the experiment with the bitter solution.

### IntelliCages

A total of 8 IntelliCages were used, with 8 to 9 animals per cage. They were placed inside a standard T2000 cage (610 × 435 × 215 mm) connected to a Type III extension cage of dimensions 425 × 266 × 155 mm to provide more living space. Food was provided *ad libitum* on a hopper on top of the cage. Saccharin solution was exchanged every 2–3 days to ensure uniform quality, quinine and plain water bottles were replaced every 4–5 days.

### Incentives and disincentives

In our study, we chose the artificial sweetener saccharin as a sweet reward, because it prevented confounding effects such as weight gain or changing body composition. We provided the animals with a 0.5% saccharin sodium salt hydrate solution (Sigma-Aldrich S1002), because we found in a previous experiment (unpublished data) that this concentration—while repulsively oversweet to the human taste—is the most attractive for mice (see also Cathomas et al., 2015). As disincentive, we used a bitter solution containing 0.3 mM quinine monohydrochloride dihydrate (Acros Organics A0420352). The approach with decreased reward probability allowed co-housing of the two groups using the same drinking bottles (4 mixed IntelliCages) and the Quinine mice were separated in 2 neutral and 2 disincentive cages.

### Protocols

*Free adaptation (FA*; *see* Figures 1B–D *for details and durations of all phases)*: First, animals were habituated to the IntelliCage environment in a free adaptation phase of 7 days, during which all doors were always open.

*Nose poke adaptation (NPA)*: Animals were introduced to the concepts of the learning tasks in these phases. During NPA I, joker side doors opened for 3 s at the beginning of every visit to a learning corner. Task side doors, on the other hand, could be opened once per visit for 3 s using a nose poke. In NPA II, the disincentive condition was introduced for the experimental groups: for the quinine group, joker side bottles were replaced with quinine solution and in the lotto group, joker side doors now immediately closed after a nose poke 75% of the time. In the next phase, NPA III, all task side bottles were replaced with sweet saccharin solution, completing the habituation to the experimental setup. NPA IV-VIII used the same protocol as NPA III and were interposed between learning tasks to reset task performance to pre-task levels.

*Place preference (PA)*: For each animal, one corner was set to be “correct,” allowing the usage of the task side, whereas the task side door remained permanently closed in all other corners. The correct corner remained constant for the entire acquisition stage and was shifted diagonally for the reversal stage. Conditions for joker sides remained the same as in NPA III. To avoid bias due to previous spontaneous preference, the most and the least preferred corners of NPA III were never assigned as correct. To avoid cage effects, correct corners were balanced as well as feasible within each cage.

*Serial reversal (SR)*: The correct corner changed every 24 h in this phase. Intentionally, a changing pattern too complex for animals to learn (shift to diagonal, then shift to long-side adjacent and so forth) was chosen to provide a pseudo-random pattern for the mice. This task did not have a reversal stage.

*Place time task (PT)*: The correct corner now moved back and forth between two adjacent corners for the entire acquisition stage, changing position in the middle of each phase of the light–dark cycle (02:00 and 14:00). In the reversal stage, the other two corners were used.

*Diagonal sequencing (DS)*: The correct corner changed diagonally after every correct task response, increasing task difficulty compared to fixed time-dependent rules. For the reversal stage, the remaining two corners were used.

*Chaining (C)*: Correct corners now changed after every task response (correct or incorrect), and now included all four corners in a clockwise or counterclockwise sequence. Direction was individually assigned to prevent imitation learning. In the reversal stage, direction was reversed.

Apart from the NPA interludes, tasks followed immediately after each other and task duration was modulated dynamically based on continuous observation of the learning curve.

### Detailed temporal analysis of door movement

In order to improve our understanding of the animals’ experience during our experiments and to define hits and hit-rates, we analyzed the exact pattern of door opening and closing by comparing raw data output from the IntelliCage with video recordings produced in a test setup without animals. We found that door opening started 0.17 s (see Supplementary Figure 2A for all timepoints) after the trigger (visit or nose poke) and the first licks were registered 0.4 s post-trigger. Doors were fully opened after 1.4 s. Closing of the door started with a latency of 0.13 s and the last licks occurred 0.7 s after the closing trigger (nose poke, automatic 3 s timer or premature end of visit). The doors were fully closed 1.37 s after the trigger and motor movement stopped shortly thereafter (1.4 s post-trigger). We observed that the first licks were registered at a time where the door position does not yet allow actual drinking—it is probable that mice prematurely started licking the metal of the bottle cap in anticipation. The last licks were registered at a door position that seemed reasonable as the last possible moment for drinking, which is why we estimated that the first moment of effective drinking occurred at this door position as well (0.9 s after opening trigger). Motor activity lasted slightly longer than the process of door movement (1.5 s).

In conclusion, with a timer setting of 3 s reward presentation (the time window allowing licks to be recorded) lasted for 3.3 s (t + 0.4 s until t + 3.7 s) and the presumed drinking window had a duration of 2.8 s (t + 0.9 s until t + 3.7 s). During analysis, hits were defined as nose pokes overlapping with reward presentation at task doors (task hits) or joker doors (joker hits). Since joker doors were programmed to accept visit onset as opening trigger, reward presentation started sooner and, sometimes, the door was already open when the first nose poke was made. This resulted in slightly reduced hit durations (Supplementary Figure 2B) and markedly reduced latencies between hitting nose pokes and licks at joker doors (Supplementary Figure 2C).

### Parameters

As the IntelliCage system’s output files only report basic variables, post-processing steps were applied to obtain composite variables such as task responses (visits to a corner with at least one nose poke on the task door), joker responses (visits with at least one nose poke on the joker door) or hits (stratified into joker and task hits).

As a measure of door preference and of motivation to engage in task leaning, we calculated the task response ratio R:


$$R=\frac{2+2*\mathrm{Task}\;\mathrm{responses}}{2+\mathrm{Joker}\;\mathrm{responses}+\mathrm{Task}\;\mathrm{responses}}$$

This value tends to 0 after many responses exclusively on the joker side and to 2 after a large number of responses exclusively on the task side. A value of 1 indicates the absence of a door preference.

As a measure of learning and task performance, we also calculated the false rate, which was defined as the percentage of task responses in incorrect corners. In the absence of a learning effect, this value is expected to be around 75%, with a significant reduction indicating successful learning of the task rule.

### Statistical analysis

Statistical analysis was performed using R software (version 4.3, used with packages ggplot2, plyr, nlme, moments, lmtest and psych). To evaluate the effect of the two methods of dual motivation, a linear model was used with two between subject factors: group (control: neutral = saccharin alone as incentive, experiment: disincentive = combined incentive-disincentive) and experiment (Quinine = quinine as disincentive, Lotto = plain water with access denied with 75% probability). The full model was set as y ~ (group*experiment*time) + Error (name), with “name” corresponding to animal ID. In the analyses shown in Supplementary Figures 2B,C, door was used instead of name. A within subject factor time was added to the model in order to explore learning effects and their dependence on group and experiment factors. Significant interactions were explored by splitting the model. Significant effects of time were further explored using partial models. Variables with strongly skewed distributions or strong correlations between variances and group means were subjected to Box-Cox transformation before statistical analysis. The significance threshold was set at 0.05. The false discovery rate (FDR) control procedure of Hochberg was applied to groups of conceptually related variables within single tests to correct significance thresholds for multiple comparisons. Similarly, FDR correction was applied during post-hoc testing. Partial ω^2^ served as measure of effect size. Comparisons of group means against chance values were performed using one-sample *t*-tests.

## Results

### Corner preference strongly established for all groups in nose poke adaptation phases

**Figure 2:**
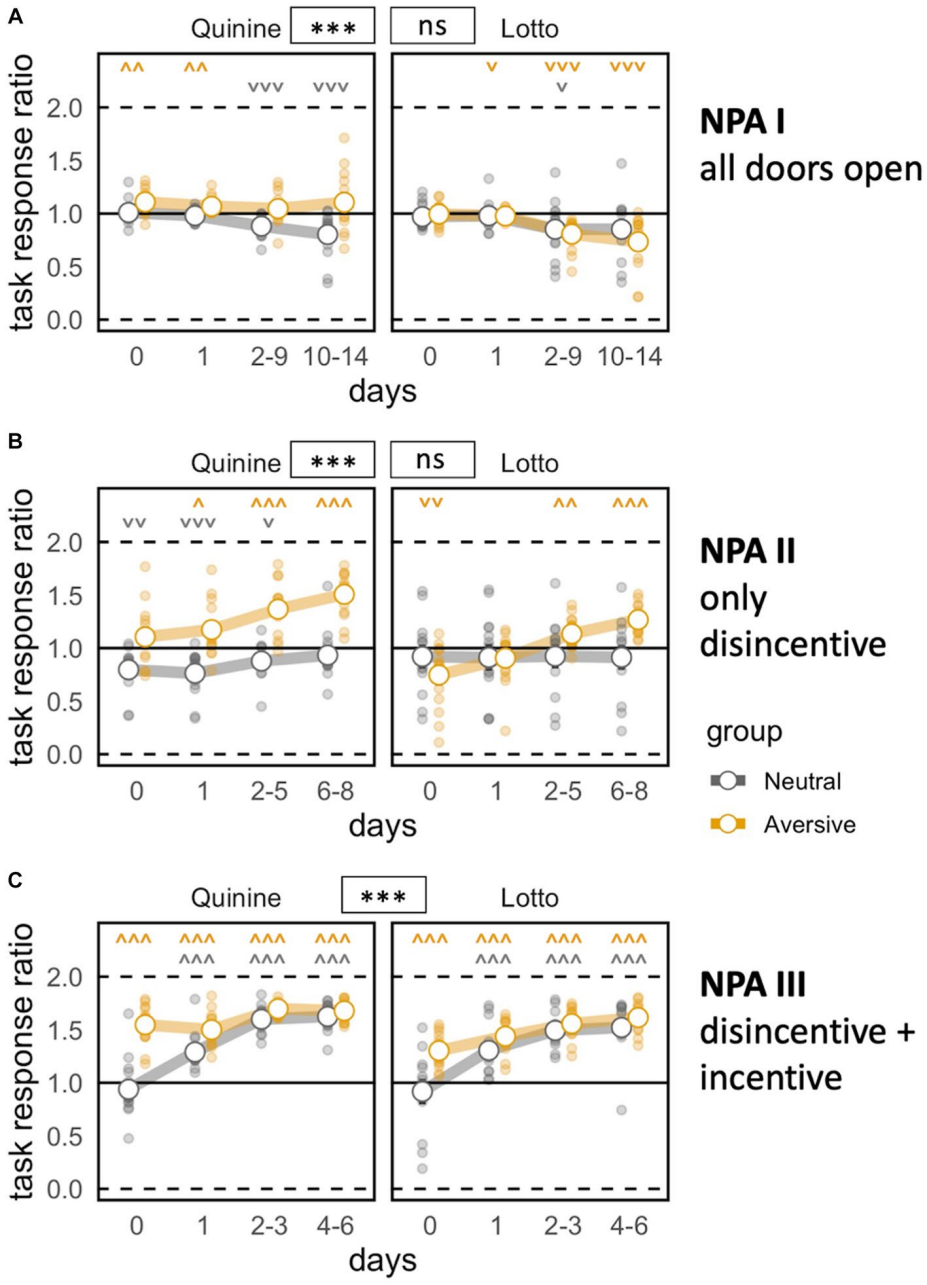
Task motivation during nose poke adaptation phases. Zero indicates total preference for joker door, 1 means no door preference, 2 means total preference for task door. ^*^p < 0.05, ^**^p < 0.01, ^***^p < 0.001 indicate group effects. **˅ ˄, ˅˅ ˄˄, ˅˅˅ ˄˄˄** stand for significant deviation from chance level according to one-sample *t*-tests. NPA, nose poke adaptation phase. Day 0 indicates last day of previous phase (pre-task baseline). **(A)** Mice develop a preference for more easily accessible joker doors except in the prospective disincentive group of Quinine experiment [time bin: *F*(3,144) = 19.22, *p* < 0.0001 ω^2^ = 0.16, group: *F*(1,48) = 7.132, *p* = 0.0103 ω^2^ = 0.11, group × time bin: bin *F*(3,144) = 0.6729 ns, group × experiment: *F*(1,48) = 33.44, *p* < 0.0001 ω^2^ = 0.39. group × experiment × time bin: *F*(3,144) = 12.36, *p* < 0.0001 ω^2^ = 0.11]. **(B)** Preference shifts to task doors upon introduction of disincentive. Experiment effect persists, probably because of pre-existing spontaneous preference [time bin: *F*(3,147) = 41.56, *p* < 0.0001, ω^2^ = 0.20, group: *F*(1,49) = 28.43, *p* < 0.0001, ω^2^ = 0.35., group × time bin: *F*(3,147) = 20.61, *p* < 0.0001, ω^2^ = 0.11, group × experiment: *F*(1,49) = 18.99, *p* < 0.0001, ω^2^ = 0.26, group × experiment × time bin: *F*(3,147) = 4.812, *p* = 0.0032, ω^2^ = 0.02]. **(C)** Control groups catch up in terms of task response ratio after incentive is introduced [time bin: *F*(3,147) = 232.3, *p* < 0.0001, ω^2^ = 0.55, group *F*(1,49) = 24.45, *p* < 0.0001, ω^2^ = 0.31, group × time bin: *F*(3,147) = 26.73, *p* < 0.0001, ω^2^ = 0.12, group × experiment *F*(1,49) = 0.1323 ns. Group × experiment × time bin: *F*(3,147) = 12.95, *p* < 0.0001, ω^2^ = 0.06].

During NPA I, a subtle but significant preference to nose poke at joker doors emerged (Figure 2A). This was likely explained by the fact that the opening of these doors was triggered by the beginning of the corner visit, giving access to water more rapidly (Supplementary Figure 2C). One prospective quinine cage showed a spontaneous preference for task doors, creating a general preference in the disincentive group of the quinine experiment. This had already been observed during free adaptation (data not shown). After the introduction of the disincentive in NPA II, preference shifted to the task door in both experimental groups (Figure 2B). The addition of saccharin to task side bottles during NPA III further increased the preference for this side in the disincentive groups, and the control groups developed a preference for the sweet liquid as well, although a small group difference persisted (Figure 2C).

### Comparable false rate, but enhanced task response ratio in place preference task

**Figure 3:**
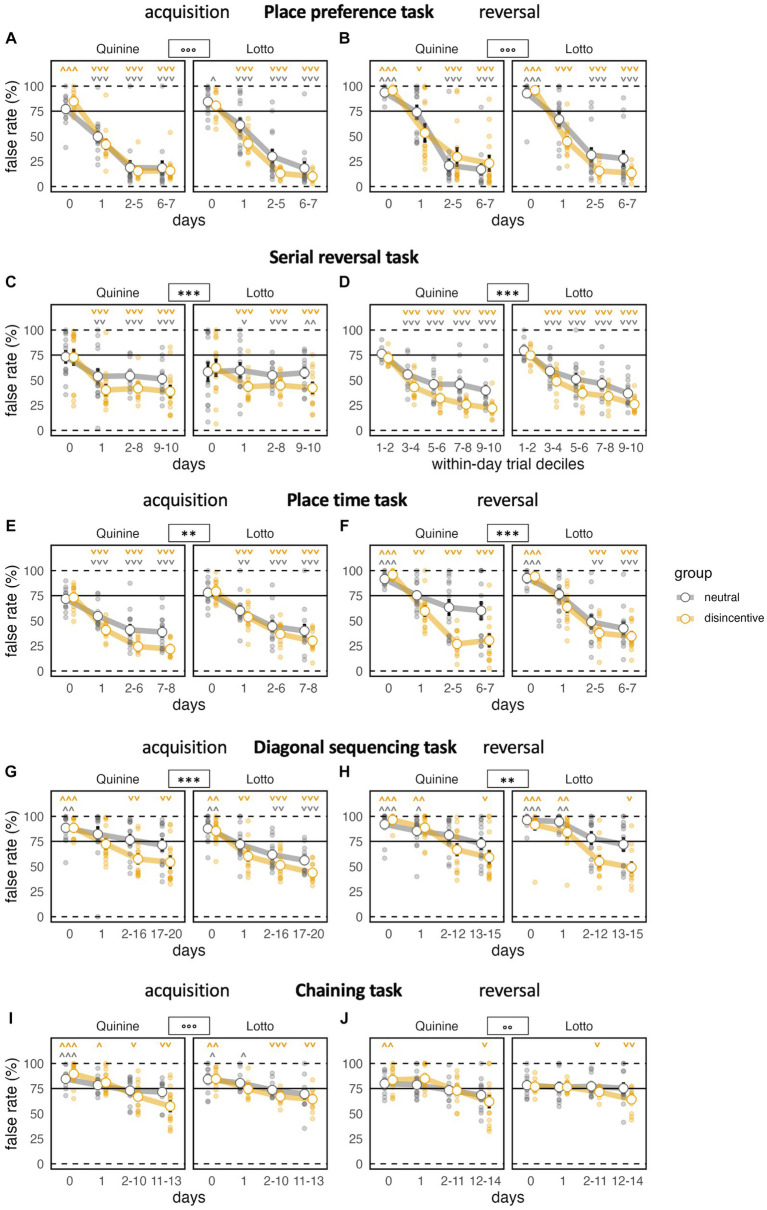
Task performance across learning phases. ^*^p < 0.05, ^**^p < 0.01, ^***^p < 0.001 indicate group effects, ° indicate group × time bin interaction (only shown when group effect not significant). **˅ ˄, ˅˅ ˄˄, ˅˅˅ ˄˄˄** stand for significant deviation from chance level according to one-sample *t*-tests. Day 0 indicates last day of previous NPA for acquisition **(A,C,E,G,I)**, last day of acquisition phase for reversal **(B,D,F,H,J)**. **(A)** All groups learn well, the disincentive group of the Lotto experiment slightly faster [time bin: *F*(3,144) = 521.0, *p* < 0.0001, ω^2^ = 0.76, group: *F*(1,48) = 3.459, *p* = 0.0690, ω^2^ = 0.05, group × time bin: *F*(3,144) = 5.852, *p* = 0.0008, ω^2^ = 0.03, group × experiment *F*(1,48) = 3.236, *p* = 0.0783, ω^2^ = 0.04, group × experiment × time bin: *F*(3,144) = 0.8052 ns]. **(B)** Again, all groups learn well, the disincentive group of the Lotto experiment slightly faster [time bin: *F*(3,141) = 280.9, *p* < 0.0001, ω^2^ = 0.72, group: *F*(1,47) = 2.787 ns, group × time bin *F*(3,141) = 6.192, *p* = 0.0006, ω^2^ = 0.05, group × experiment: *F*(1,47) = 2.276 ns, group × experiment × time bin: *F*(3,141) = 2.465, *p* = 0.0649, ω^2^ = 0.01]. **(C)** Learning is intact, but not progressive after first day in serial reversal (SR) task. Disincentive groups perform better, irrespective of experiment [time bin: *F*(3,138) = 10.57, *p* < 0.0001, ω^2^ = 0.14, group: *F*(1,46) = 12.76, *p* = 0.0008, ω^2^ = 0.20, group × time bin: *F*(3,138) = 1.833 ns, group × experiment: *F*(1,46) = 0.1020 ns, group × experiment × time bin: *F*(3,138) = 0.2525 ns]. **(D)** Progressive performance in serial reversal task when analyzed by block bins, which correspond to deciles of numbers of trials within a day/task. Disincentive groups perform better and learn faster, irrespective of experiment [block bin: *F*(1,220) = 663.8, *p* < 0.0001 ω^2^ = 0.61, group: *F*(1,46) = 27.14, *p* < 0.0001, ω^2^ = 0.35, group × block bin: *F*(1,220) = 9.778, *p* = 0.0020, ω^2^ = 0.02, group × experiment: *F*(1,46) = 0.3550 ns, group × experiment × block bin: *F*(1,220) = 0.6738 ns]. **(E)** Good performance in all groups, disincentive groups learn faster and perform better [time bin: *F*(3,138) = 323.2, *p* < 0.0001, ω^2^ = 0.67, group: *F*(1,46) = 9.302, *p* = 0.0038, ω^2^ = 0.15, group × time bin: time bin *F*(3,138) = 9.999, *p* < 0.0001, ω^2^ = 0.05, group × experiment: *F*(1,46) = 1.521 ns, group × experiment × time bin: *F*(3,138) = 0.1349 ns]. **(F)** All groups learn the reversal stage with modest performance of controls in the Quinine experiment. Again, disincentive groups learn faster and perform better [time bin: *F*(3,138) = 153.4, *p* < 0.0001, ω^2^ = 0.61, group: *F*(1,46) = 18.40, *p* < 0.0001, ω^2^ = 0.27, group × time bin: *F*(3,138) = 10.51, *p* < 0.0001, ω^2^ = 0.09, group × experiment: *F*(1,46) = 1.011 ns, group × experiment × time bin: *F*(3,138) = 1.941 ns]. **(G)** All groups learn, starting off above chance level. Disincentive groups learn faster and perform better. False rate of controls in the Lotto experiment does not fall significantly below chance level [time bin: *F*(3,138) = 80.43, *p* < 0.0001, ω^2^ = 0.40, group: *F*(1,46) = 10.44, *p* = 0.0023, ω^2^ = 0.16, group × time bin: *F*(3,138) = 4.488, *p* = 0.0049, ω^2^ = 0.03, group × experiment: *F*(1,46) = 0.0192 ns, group × experiment × time bin: *F*(3,138) = 0.1339 ns]. **(H)** All groups improve, starting clearly above chance level. Disincentive groups learn faster and perform better. False rate of controls in both experiments fails to fall significantly below chance level [time bin: *F*(3,138) = 91.22, *p* < 0.0001, ω^2^ = 0.42, group: *F*(1,46) = 7.935, *p* = 0.0071, ω^2^ = 0.13, group × time bin: *F*(3,138) = 8.974, *p* < 0.0001, ω^2^ = 0.06, group × experiment: *F*(1,46) = 1.628 ns, group × experiment × time bin: *F*(3,138) = 0.0904 ns]. **(I) S**tarting above chance level, all groups still improve, but more slowly than in previous tasks. Disincentive groups learn faster while control groups fail to improve significantly below chance level [time bin: *F*(3,132) = 80.11, *p* < 0.0001, ω^2^ = 0.39, group: *F*(1,44) = 2.541 ns, group × time bin: *F*(3,132) = 7.810, *p* < 0.0001, ω^2^ = 0.05. group × experiment: *F*(1,44) = 1.086 ns group × experiment × time bin: *F*(3,132) = 3.4 28, *p* = 0.0191, ω^2^ = 0.02]. **(J)** Learning is overall slower than during acquisition. Disincentive groups learn slightly faster and unlike controls reach a final false rate significantly below chance [time bin: *F*(3,132) = 25.33, *p* < 0.0001, ω^2^ = 0.19, group: *F*(1,44) = 0.5358 ns, group × time bin: *F*(3,132) = 5.160, *p* = 0.0021, ω^2^ = 0.04, group × experiment: *F*(1,44) = 0.6728 ns, group × experiment × time bin: *F*(3,132) = 0.2386 ns].

**Figure 4:**
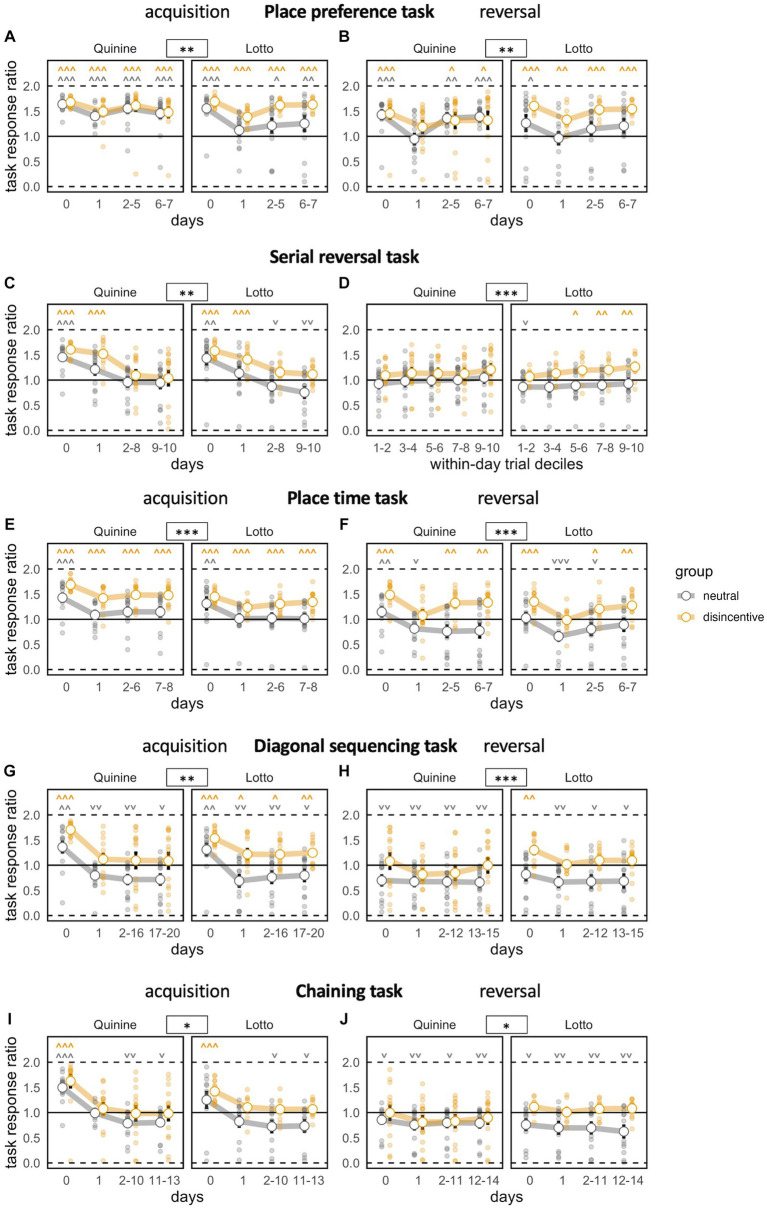
Task motivation across learning phases. Zero indicates total preference for joker door, 1 means no door preference, 2 means total preference for task door. ^*^p < 0.05, ^**^p < 0.01, ^***^p < 0.001 indicate group effects. **˅ ˄, ˅˅ ˄˄, ˅˅˅ ˄˄˄** stand for significant deviation from chance level according to one-sample *t*-tests. Day 0 indicates last day of previous NPA for acquisition **(A,C,E,G,I)**, last day of acquisition phase for reversal **(B,D,F,H,J)**. **(A)** Preference to respond at task doors decreases transiently as task begins. Controls, especially of the Lotto experiment, show weaker preference to respond at task doors [time bin: *F*(3,144) = 45.21, *p* < 0.0001, ω^2^ = 0.25, group: *F*(1,48) = 9.446, *p* = 0.0035, ω^2^ = 0.14, group × time bin *F*(3,144) = 0.7165 ns, group × experiment: *F*(1,48) = 3.566, *p* = 0.0650, ω^2^ = 0.05, group × experiment × time bin: *F*(3,144) = 0.7024 ns]. **(B)** Preference to respond at task doors decreases transiently as target corner changes. Controls, especially of the Lotto experiment, show weaker preference to respond at task doors [time bin: *F*(3,141) = 39.05, *p* < 0.0001, ω^2^ = 0.16, group: *F*(1,47) = 5.789, *p* = 0.0201, ω^2^ = 0.09, group × time bin: *F*(3,141) = 0.4827 ns, group × experiment: *F*(1,48) = 3.566, *p* = 0.0650, ω^2^ = 0.05, group × experiment: × time bin: *F*(3,141) = 1.548 ns]. **(C)** Task response ratio decreases persistently, to levels slightly above chance in disincentive groups, to levels slightly below chance in controls [time bin: *F*(3,138) = 82.57, *p* < 0.0001, ω^2^ = 0.43, group: *F*(1,46) = 11.46, *p* = 0.0015, ω^2^ = 0.18, group × time bin: *F*(3,138) = 1.069 ns, group × experiment: *F*(1,46) = 0.0008 ns, group × experiment × time bin: *F*(3,138) = 0.5364 ns]. **(D)** Progressive increase in motivation in serial reversal task when analyzed by block bins, which correspond to deciles of numbers of trials within a day/task. Only the disincentive group of the Lotto experiment develops a significant preference to respond at task doors [block bin: *F*(1,220) = 102.8, *p* < 0.0001, ω^2^ = 0.03, group: *F*(1,46) = 7.317, *p* = 0.0095, ω^2^ = 0.12, group × block bin: *F*(1,220) = 8.082, *p* = 0.0049, ω^2^ = 0.00, group × experiment: *F*(1,46) = 0.1098 ns, group × experiment × block bin: *F*(1,220) = 5.600 *p* = 0.0188 ω^2^ = 0.00]. **(E)** Task response ratio decreases persistently as task begins. Only disincentive groups maintain a significant preference to respond at task doors [time bin: *F*(3,138) = 63.50, *p* < 0.0001, ω^2^ = 0.25, group: *F*(1,46) = 25.28, *p* < 0.0001, ω^2^ = 0.34, group × time bin: *F*(3,138) = 1.685 ns, group × experiment: *F*(1,46) = 1.439 ns, group × experiment × time bin: *F*(3,138) = 0.9273 ns]. **(F)** Task response ratio drops further as the rule is reversed, followed by partial recovery. While disincentive groups reestablish preferential responding at task doors, controls transiently prefer to respond at joker doors [time bin: *F*(3,138) = 34.97, *p* < 0.0001, ω^2^ = 0.19, group: *F*(1,46) = 32.07, *p* < 0.0001, ω^2^ = 0.39, group × time bin: *F*(3,138) = 2.420, *p* = 0.0688, ω^2^ = 0.01, group × experiment: *F*(1,46) = 0.4442 ns, group × experiment × time bin: *F*(3,138) = 0.1100 ns]. **(G)** Task response ratio markedly and persistently decreases as task begins. Controls prefer to respond at joker doors throughout the task, while controls slightly favor responding at task doors [time bin: *F*(3,138) = 72.26, *p* < 0.0001, ω^2^ = 0.31, group: *F*(1,46) = 21.02, *p* < 0.0001, ω^2^ = 0.29, group × time bin: *F*(3,138) = 1.134 ns, group × experiment: *F*(1,46) = 1.108 ns, group × experiment × time bin: *F*(3,138) = 0.9775 ns]. **(H)** Task response ratio decreases further as the task rule is reversed. Controls consistently prefer to respond at joker doors, while controls respond near chance level [time bin: *F*(3,138) = 6.490, *p* = 0.0004, ω^2^ = 0.03, group: *F*(1,46) = 12.68, *p* = 0.0009, ω^2^ = 0.20, group × time bin: *F*(3,138) = 1.786 ns, group × experiment: *F*(1,46) = 1.585 ns, group × experiment × time bin: *F*(3,138) = 1.023 ns]. **(I)** Task response ratio strongly decreases as task begins, without recovery. Controls prefer to respond at joker doors, while controls respond near chance level [time bin: *F*(3,132) = 102.7, *p* < 0.0001, ω^2^ = 0.33, group: *F*(1,44) = 5.754, *p* = 0.0208, ω^2^ = 0.09, group × time bin *F*(3,132) = 1.133 ns, group × experiment: *F*(1,44) = 0.2809 ns, group × experiment × time bin: *F*(3,132) = 0.3633 ns]. **(J)** Motivation further decreases and shows a similar pattern as in acquisition [time bin: *F*(3,132) = 9.649, *p* < 0.0001, ω^2^ = 0.02, group: *F*(1,44) = 6.945, *p* = 0.0116, ω^2^ = 0.11, group × time bin: *F*(3,132) = 2.637, *p* = 0.0523, ω^2^ = 0.00, group × experiment: *F*(1,44) = 0.2784 ns, group × experiment × time bin: *F*(3,132) = 1.153 ns].

In the acquisition stage, we found a strong decrease in the percentage of false corner choices compared to baseline, where the correct corner was not yet noticeably different. In this phase, neither disincentive group showed a false corner choice rate that was significantly better than the controls. However, a group x time bin interaction effect revealed a somewhat steeper learning curve for the disincentive groups (Figure 3A). Task response ratio was significantly higher in disincentive groups, as well (Figure 4A). While graphs suggested a somewhat stronger effect in the Lotto group, this remained below the threshold of significance.

During the reversal stage, false rates dropped sharply and significantly, but did not fully reach the levels of the acquisition phase. The patterns of the acquisition phase were replicated, with no significant difference in false rates, but significant effects on improvement rate and task response ratio (Figures 3B 4B).

### Improved learning in serial reversal task

In this task, we saw a significantly reduced false rate in both groups compared to baseline, with better performance in disincentive groups, but no evidence for a difference between the two disincentive groups (Figure 3C). False rates after baseline reached a plateau and did not further improve across days, which indicates that the mice, as expected, did not understand the corner change pattern and learned each target position as a new task. Task response ratio fell significantly across time bins. However, disincentive groups still showed a significantly higher task response ratio than controls (Figure 4C).

When trials within days (where the same corner remained correct) were grouped into block bins (corresponding to deciles of the trial number within that time period), false rates steeply declined for all groups, with disincentive groups again displaying more robust learning (Figure 3D). Block bin analysis also showed that overall task response ratio steadily and significantly, but slowly increased within days, with higher levels in disincentive groups (Figure 4D).

### Intact learning in place time task with stronger performance of disincentive groups

False rates dropped significantly from chance levels at baseline. The fact that false rates continued to fall after the implementation of the task rule across time bins showcases the animals’ ability to understand the simpler back-and-forth change pattern employed here (Figure 3E). While disincentive groups performed better, a potential trend toward stronger learning in the Quinine group compared to the Lotto group remained not significant.

Task response ratio fell significantly after the introduction of the task rule, but remained stronger in the disincentive groups (Figure 4E).

During reversal, the pattern of the acquisition phase was mostly replicated, with significant effects of time bin and disincentive group and a non-significant trend toward stronger performance in the Quinine group compared to the Lotto group (Figures 3F 4F).

### Diagonal sequencing with only disincentive groups retaining above-chance task response ratio

False rates dropped significantly from chance levels at baseline and we again saw a steady decline in false rate, which was expected in a task with a constant (or rather, constantly changing) task rule. However, false rates did not reach the levels seen in the previous phases, mirroring the increased difficulty of the task. Groups showed a similar pattern as in the previous phase, with better performance of disincentive groups (Figure 3G).

Task response ratio fell significantly after the introduction of the task rule, but remained stronger in the disincentive groups. It should be noted that task response ratio only remained above chance for disincentive groups (Figure 4G).

In the reversal stage, false rates still dropped, but not as strongly as in the acquisition phase. While all groups started clearly above chance levels (because baseline was recorded under the acquisition task rule), only disincentive groups were able to reach levels below chance (Figure 3H). Task response ratio was also lower than in the acquisition phase. While disincentive groups never fell below chance levels, controls were always below chance, meaning they preferred to reliably receive plain water at joker doors (Figure 4H).

### Disincentive groups with preserved learning performance into chaining task

False rates in the acquisition phase were overall higher than in the diagonal sequencing task, reflecting the fact that difficulty increased yet again. Here, the false rates of disincentive groups were no longer lower than in controls, but there was a significant interaction between group and time bin, indicating a steeper learning curve in disincentive groups. In this phase, there was also a significant interaction between time bin, group and disincentive type, with a steeper learning curve in the Quinine experiment (Figure 3I). Task response ratio decreased over time, more strongly in control groups. Controls fell below chance levels (Figure 4I).

During chaining reversal, false rates overall were the highest recorded in any phase, but the decline across time bins was still significant. Again, false rate in disincentive groups was no longer reduced, but their learning curves were significantly steeper. Task response ratios were also low throughout this phase, but significant changes were noticeable for time bins and group (Figures 3J 4J).

### Lick numbers and task motivation decreased across phases, but were increased in disincentive groups

When comparing the number of total licks per day across phases, we found that values decreased over the course of the study both for nose poke adaptation and learning phases. After an initial drop, numbers stabilized at a level of approximately 1,000 licks per day. There was no evidence for decreased lick numbers in disincentive groups compared to controls (Supplementary Figures 1B,D).

Task response ratio also dropped markedly across phases in control as well as in disincentive groups. In learning tasks (Supplementary Figure 1C), this can be explained by increasing task difficulty, but the decrease in the nose poke adaptation phases (Supplementary Figure 1A) also shows an overall loss of motivation. Task response ratio remained significantly enhanced for disincentive groups during NPA as well as learning phases.

## Discussion

Our findings confirm and expand the results of previous studies that examined reward learning in IntelliCage (Bramati et al., 2023). The combination of incentive and disincentive resulted in an overall stronger motivation to learn, which is reflected by consistently higher task response ratio and results in better performance and/or higher learning rate, as well as the preservation of the learning effect even into more difficult hippocampus-dependent learning tasks.

However, when comparing our study to IntelliCage experiments based on drinking restrictions, it appears that our approach still elicited a somewhat weaker learning response in difficult learning tasks: For instance, animals in previous studies (Kobayashi et al., 2013; Akbergenov et al., 2018; Mätlik et al., 2018; van Dijk et al., 2019) were all able to deliver false rates of 30–40% on average in the most difficult task used in this study (chaining), which exceeds our results of around 60% in this phase. On the other hand, a recently published study using captured wild rodents (Jörimann et al., 2023) found chaining phase false rates that were roughly comparable to ours.

Future studies using our protocols would not necessarily need to include the most difficult tasks used here. Depending on the research question, simpler memory tasks such as diagonal sequencing or the place time task may already suffice. However, it is important to always include a very simple task such as place preference (PP) to check the intactness of basal sensorimotor functions, as it is usually done in classical Morris Water Maze testing by adding a much easier cue-based version as control (Vorhees and Williams, 2006).

Even though task motivation as measured by task response rates was consistently improved by the use of our dual-motivation protocols, these protocols could not prevent a decline of task engagement with increasing task difficulty. Task engagement and also total liquid consumption measured by lick number deteriorated over the course of our study even when examining the interposed nose poke adaptation phases. This suggests that a certain habituation effect was present. Animals might have lost their initial fascination with the sweet taste stimuli and increasingly limited their efforts to the minimum required to prevent dehydration. Possible ways to address this could include a quicker progression to more difficult learning tasks or the replacement of saccharin solution with plain water during nose poke adaptation interludes. The latter would provide a sensation of novelty once learning resumes and could condition the mice to strictly associate sweet rewards with task completion.

A potential limitation of our study lies in the fact that we exclusively tested female animals. In a previous experiment (Nigri et al. submitted to the same special issue of Frontiers in Behavioral Neuroscience), we observed that females show a more robust motivation to learn reward-learning tasks (see also Chen et al., 2021, for sex differences in reward learning) and thus, it remains to be shown whether our protocol sufficiently motivates males. If learning is unsatisfactory in this case, the paradigm could be further escalated by combining the two disincentives, simultaneously decreasing the chance for access to joker doors and replacing their bottle content with quinine solution.

Aside from potentially replacing traditional thirst-based IntelliCage learning protocols, adapted versions of our protocols could also be used to investigate specific effects of genetic modifications or pharmacological compounds on reward learning as compared to thirst-driven learning. For instance, many neurodegenerative diseases have specific impacts on the sensitivity to reward (see Perry and Kramer, 2015, for a review on the topic). Combined protocols could help to describe deficient behavioral phenotypes more specifically. Perhaps, some mouse models might also show impairments in reward learning experiments that would have remained masked under the binary task rules of conventional protocols.

We found no consistent differences in efficacy between the two disincentives. However, both have some advantages and disadvantages. The Lotto approach allows co-housing of both groups, which eliminates the possibility of cage effects. During the nose poke adaptation phases, the emergence of such a cage effect in of the quinine IntelliCages complicated analysis to a certain degree. On the other hand, using quinine as the disincentive limits the paradigm to taste preference alone, which facilitates interpretation on a neurological level. Meanwhile, the Lotto group was exposed to a combination of an attractive taste stimulus and a disincentive, which could best be described as an unattractive low reward probability/low reward value gambling task (Pittaras et al., 2020). This complicates the underlying cognitive mechanisms and, consequently, the interpretation of findings from such an experiment series.

In conclusion, we demonstrated that a combined approach of positive and negative drivers can be used to provide motivation even for complex learning tasks, which stands in contrast to the more rapidly waning effect of positive/appetitive motivation alone. Importantly, the introduction of the disincentives did not lead to a reduction in the number of licks and thus did not expose animals to the risk of dehydration. The protocols we described can be used to replace conventional spatial learning tasks that rely on drinking restrictions, improving animal welfare. However, our study also highlights the limitations of this approach. Even with the improved paradigm presented here, our results suggest a somewhat weaker learning performance than seen in conventional approaches. Because of this, more research in this direction is needed to further exploit the vast possibilities of modified IntelliCage protocols in the service of animal welfare.

## Data availability statement

The raw data supporting the conclusions of this article will be made available by the authors, without undue reservation.

## Ethics statement

The animal study was approved by Cantonal Veterinary Office of Zurich (License No. 060/2021). The study was conducted in accordance with the local legislation and institutional requirements.

## Author contributions

DW: design and concept of the study and statistical analysis. XM, BS, and IA: mouse behavioral phenotyping. DW, AS, and IA: local support and coordination with planning, protocols, equipment and animal orders. XM, BS, AS, IA, MN, GB, and DW: discussing the data. AS: writing the manuscript and preparing the figures. All authors contributed to the article and approved the submitted version.
