# Supplementary figures and images for: Refinement of IntelliCage protocols for complex cognitive tasks through replacement of drinking restrictions by incentive-disincentive paradigms
Source: Front Behav Neurosci. 2023 Nov 16;17:1232546. doi: 10.3389/fnbeh.2023.1232546 (PMC10687469; doi:10.3389/fnbeh.2023.1232546)

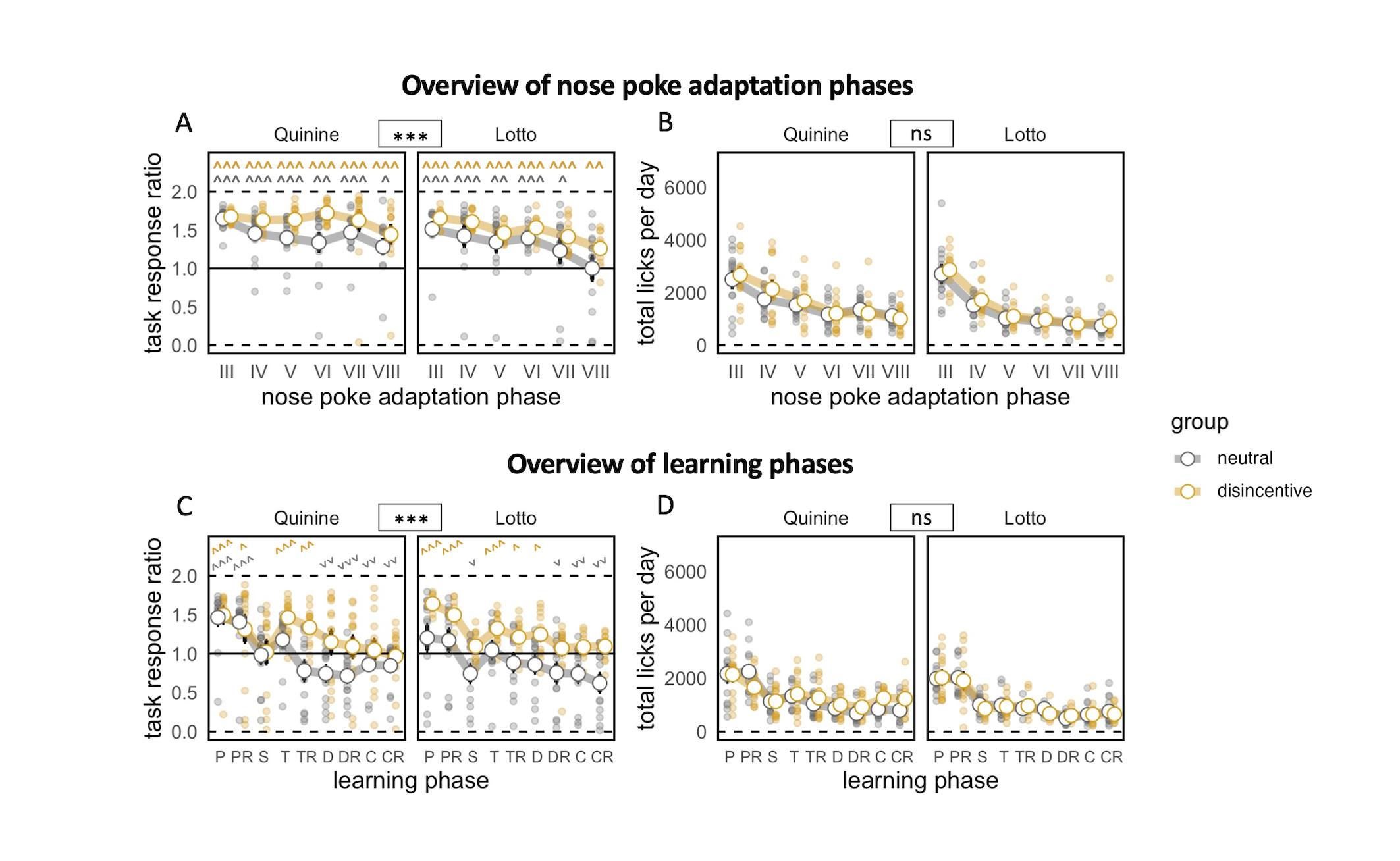

Supplement: Supplementary file 1 [file Image_1.TIFF]

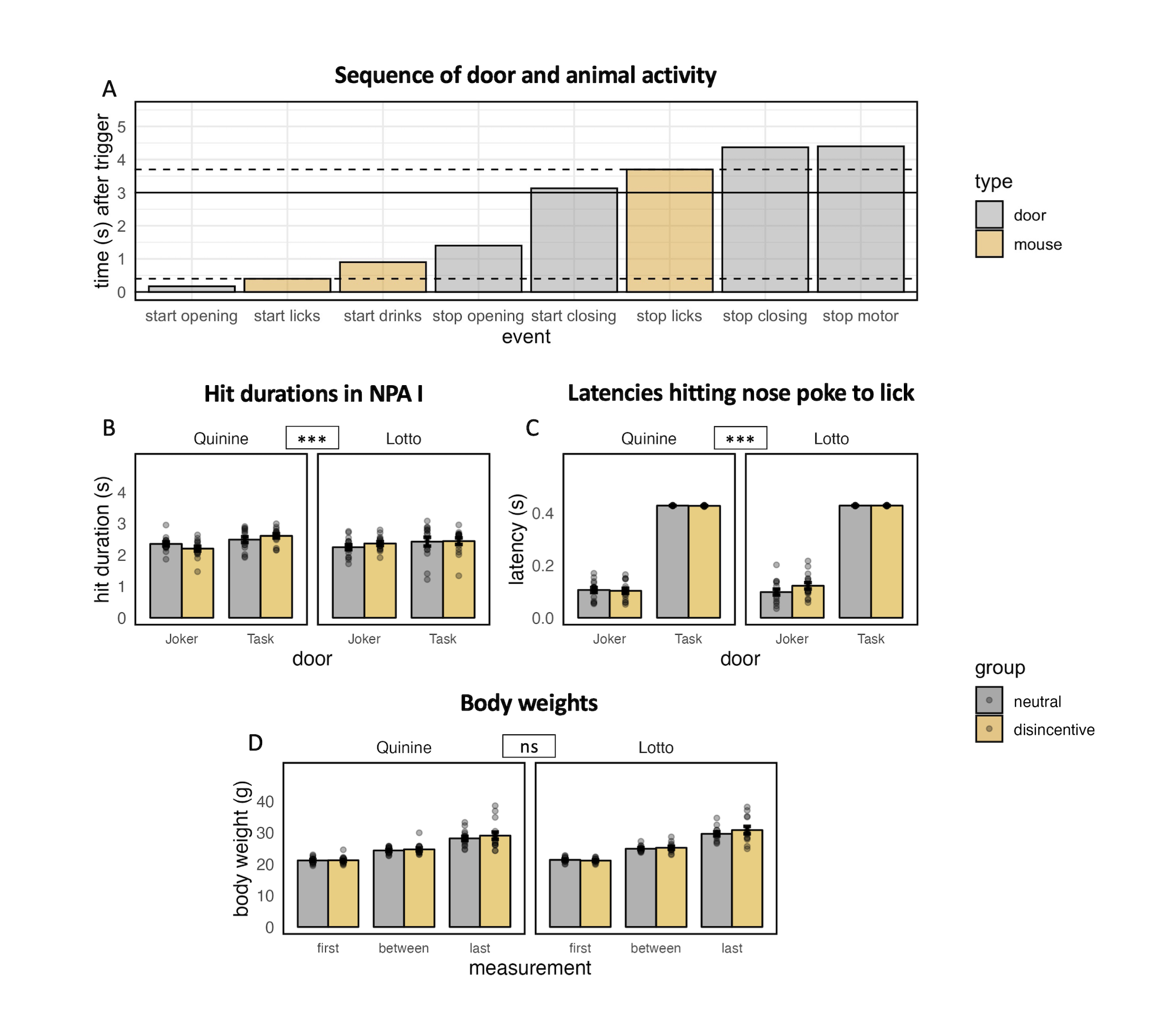

Supplement: Supplementary file 2 [file Image_2.TIFF]
